# Fate of the Left Pulmonary Artery and Thoracic Aorta After Transcatheter Patent Ductus Arteriosus Closure in Low Birth Weight Premature Infants

**DOI:** 10.1007/s00246-020-02523-8

**Published:** 2021-01-04

**Authors:** Dor Markush, Jennifer C. Tsing, Surbhi Gupta, Nicole C. Berndsen, Geena Radville, Ruchira Garg, Evan M. Zahn, Myriam Almeida-Jones

**Affiliations:** 1 Guerin Family Congenital Heart Program, Smidt Heart Institute, Cedars-Sinai Medical Center, Los Angeles, CA USA; 2 Department of Pediatrics, Cedars-Sinai Medical Center, Los Angeles, CA USA; 3 Department of Pediatrics, University of California Los Angeles, Los Angeles, CA USA; 4 University of Texas at Austin, Austin, TX USA; 5 Guerin Family Congenital Heart Program, Cedars-Sinai Smidt Heart Institute, 127 S. San Vicente Blvd, Suite A3600, Los Angeles, CA 90048 USA

**Keywords:** Patent ductus arteriosus, Premature, Transcatheter closure, Vascular obstruction

## Abstract

Transcatheter patent ductus arteriosus closure (TCPC) is an emerging treatment for low birth weight extremely premature neonates (EPNs). Left pulmonary artery (LPA) and descending aorta (DAO) obstruction are described device-related complications, however, data on mid- and long-term vascular outcomes are lacking. A retrospective analysis of EPNs who underwent successful TCPC at our institution from 03/2013 to 12/2018 was performed. Two-dimensional echocardiography and spectral Doppler velocities from various time points before and after TCPC were used to identify LPA and DAO flow disturbances. A total of 44 EPNs underwent successful TCPC at a median (range) procedural weight of 1150 g (755–2500 g). Thirty-two (73%) patients were closed with the AVP II and 12 (27%) with the Amplatzer Piccolo device. LPA and DAO velocities on average remained within normal limits and improved spontaneously in long-term follow up (26.1 months, range 1–75 months). One patient, who had concerning LPA flow characteristics immediately after device implant (peak velocity 2.6 m/s) developed progressive LPA stenosis requiring stent placement 3 months post-procedure. In the remaining infants, including 7 (16%) who developed LPA and 3 (7%) who developed DAO flow disturbances (range 2–2.4 m/s), all had progressive normalization of flow velocities over time. TCPC can be performed safely in EPNs with a low incidence of LPA and DAO obstruction. In the absence of significant progressive vascular obstruction in the early post-procedure period, mild increases in LPA and DAO flow velocities tend to improve spontaneously and normalize in long-term follow-up.

## Introduction

Closure of the patent ductus arteriosus (PDA) is often delayed in extremely premature neonates (EPNs), with a higher risk for developing a hemodynamically significant PDA (hsPDA) associated with lower gestational age [1]. A hsPDA results in a large left-to-right shunt with aortic diastolic runoff, the presence of which has been associated with significant morbidity and mortality in this population [2–4]. While a direct causal relationship between a hsPDA and several important premature neonatal comorbid conditions such as necrotizing enterocolitis and bronchopulmonary dysplasia has not been fully established [5], the higher risk associated with a hsPDA in EPNs often prompts treatment [6]. Historically, pharmacological therapy and/or surgical ligation have been the only alternatives to medical management of a hsPDA. As each is associated with its own set of short- and long-term complications [7–11], this has led investigators to develop alternative strategies for managing hsPDA in EPNs.

Transcatheter patent ductus arteriosus closure (TCPC) is the treatment of choice for PDA closure in infants ≥ 6 kg, older children, and adults [12, 13]. More recently, the feasibility and success of TCPC have been demonstrated in EPNs, even those with procedural weight of < 1000 g [14–18]. Previous concerns related to vascular access and device positioning have been largely addressed with advances in catheter and device technology and the development of new techniques specifically designed for the treatment of hsPDA in this unique population [15, 19–21]. Important procedural complications reported to date include the development of left pulmonary artery (LPA) or descending aorta (DAO) stenosis, device embolization, tricuspid valve damage, and traumatic injury to the inferior vena cava [14, 22, 23]. Recent concerns have also emerged regarding the potential for delayed onset LPA or DAO obstruction [15, 24]. As TCPC in EPNs is a newly described procedure, a paucity of data exists on mid- and long-term outcomes related to vascular stenosis. The aim of our study was to describe LPA and DAO echo-derived flow patterns in EPNs following TCPC, and to characterize the early and long-term risk for development of LPA and/or DAO obstruction.

## Methods

### Study Population

This is a retrospective analysis of EPNs who underwent TCPC between March 2013 and December 2018 at our institution. We included all EPNs who underwent successful TCPC—defined as leaving the catheterization laboratory after implantation of a PDA closure device—which was performed during their initial NICU hospitalization, and who were > 6 months out from their procedure at the time of data collection. Patients excluded from this analysis were those in whom TCPC was attempted but aborted before device release due to technical issues or concerns of vascular obstruction as described in the procedural and echocardiographic details below, and those with known preexisting LPA or DAO obstruction prior to TCPC.

Data collected included birth weight and gestational age (GA), sex, age and weight at time of catheterization, procedural location (cardiac catheterization laboratory versus neonatal intensive care unit), access choice, ductal diameters and characteristics, device type and size, fluoroscopy and procedure times, associated medical comorbidities, and echocardiographic parameters. The study was approved by the Cedars-Sinai Medical Center IRB (CS IRB #PRO53442).

### Procedural Details

We have previously described our technique for TCPC in EPNs [14, 20]. All implants were performed from a transvenous approach and in every case efforts were made to avoid femoral arterial entry. Earlier in our experience, the Amplatzer Vascular Plug II (AVP II, Abbott Structural Heart, Minneapolis, MN) was the primary device utilized for TCPC in EPNs [14, 20]. However, more recently the Amplatzer Ductal Occluder II Additional Sizes (ADO II-AS), recently renamed the Amplatzer Piccolo device (Abbott Structural Heart, Minneapolis, MN), has been used exclusively. Procedures were performed either in the NICU or the cardiac catheterization suite, based upon operator discretion. Regardless of procedural location, device implantation was performed using a combination of fluoroscopic and echocardiographic guidance. Following device deployment, prior to device release, the LPA and DAO were evaluated for evidence of device-related obstruction using a combination of 2-dimensional (2D) imaging and color/spectral Doppler performed by a single experienced imager (RG). If questions remained about obstruction to LPA flow, a small hand angiogram was performed via the delivery catheter prior to device release. The presence or absence of DAO obstruction was based solely on echocardiographic assessment. If device-related vascular stenosis was suspected, the device was repositioned and/or recaptured and replaced. If stenosis persisted despite these maneuvers, the device was removed, the procedure aborted, and the patient converted to surgical ligation (such patients are excluded from the current analysis).

### Echocardiography and Follow-Up

EPNs referred for TCPC underwent extensive transthoracic echocardiographic evaluation at the following intervals: (1) pre-procedure, (2) intra-procedure, (3) early post-procedure (< 24 h), (4) 1–3 months post-procedure, and yearly thereafter. All patients included were at least 6 months post-procedure. Seven patients did not have long-term follow up available due to being transferred from other institutions or having relocated. For these patients, latest post-TCPC imaging available (all at least 1 month out) was recorded as most recent follow-up. Following the procedure, each echocardiographic evaluation included detailed evaluation of device location and positioning, residual ductal shunting, the presence or absence of neighboring vascular obstruction, tricuspid regurgitation, and left ventricular size (2D end-diastolic dimension, and volume by area-length method) and function. Echocardiograms were performed per our institution’s protocol for PDA assessment pre and post-TCPC in premature neonates, and in accordance with guidelines from the American Society of Echocardiography [25]. LPA and DAO stenosis by echocardiography was defined as 2D evidence of vessel luminal narrowing in the context of increased peak Doppler velocity (> 2 m/s, estimating instantaneous gradient > 16 mmHg) and/or an obstructed flow pattern (persistent antegrade diastolic flow).

### Statistical Analysis

Continuous variables are expressed as mean ± standard deviation or median (range). Paired student t-tests were used to compare continuous variables, including LPA and DAO Doppler velocities. Statistical analysis was performed using IBM SPSS Statistics for Windows, version 24.0 (Armonk, NY). A *p* value of < 0.05 was considered statistically significant.

## Results

### Patient Demographics

A total of 49 neonates underwent attempted TCPC, of which 45 (92%) had successful closure. In 4 patients the procedure was aborted secondary to risk of device-related LPA obstruction (3 patients) or inability to advance the delivery sheath across the PDA due to ductal spasm (1 patient). All 4 aborted procedures took place earlier in our experience using the AVP II. One severely ill 1200 g EPN underwent successful TCPC in the setting of multiple muscular ventricular septal defects and recognized native aortic coarctation (CoA). This patient subsequently required aortic stenting for the native CoA in the context of prematurity and ultimately underwent successful surgical repair of CoA. The remaining 44 patients constitute our study group.

Patient demographic data are shown in Table 1. The majority of subjects (89%) were born at ≤ 28 weeks gestational age and 95% with a birthweight < 1500 g. A high percentage (91%) had significant lung disease requiring either conventional or high frequency oscillator mechanical ventilation before their procedure. Most patients (70%) had an unsuccessful attempt at pharmacologic PDA closure. Several neonates had significant comorbidities related to prematurity, including 11 (24%) who had necrotizing enterocolitis and 7 (16%) with ≥ Grade II intraventricular hemorrhage.

**Table 1:** Patient demographics

Birth weight	848 (440–2480) g
≤ 2500 g	44 (100%)
≤ 1500 g	42 (95%)
≤ 1000 g	30 (68%)
Birth gestational age	26.4 (23.6–33.3) weeks
23–25 weeks	17 (39%)
26–28 weeks	22 (50%)
29–31 weeks	3 (7%)
≥ 32 weeks	2 (4%)
Female	20 (46%)
PDA pharmacological therapy attempted	31 (70%)
Mechanical ventilation requirement	
SIMV	28 (64%)
HFOV	12 (27%)
NIPPV	1 (2%)
NC	1 (2%)
RA	2 (5%)

Data expressed as median (range), or number (%)

*SIMV* synchronized intermittent mechanical ventilation, *HFOV* high frequency oscillatory ventilation, *NIPPV* non-invasive positive pressure ventilation, *NC* nasal canula, *RA* room air

### Procedural Data

Procedural data are summarized in Table 2. All patients were ≤ 2.5 kg at the time of TCPC (median 1.1 kg), with 89% of patients being ≤ 2 kg and 30% having a procedural weight < 1 kg. Based largely on timing of the study period, the majority of PDAs in this series (73%) were closed with the AVP II device and 27% with the Piccolo. Transvenous device delivery was used in all cases, with arterial access also obtained in 2 patients (4%). Average post-procedure imaging follow-up was 2.2 years with a range of 1–75 months.

**Table 2:** Procedural data

Weight	1152 (755–2500) g
≤ 2.5 kg	44 (100%)
≤ 2 kg	39 (89%)
≤ 1 kg	13 (30%)
Age	27 (5–76) days
Corrected gestational age	29.6 (27.2–40.3) weeks
Procedural location (cath lab)	31 (71%)
Venous access only	42 (96%)
PDA minimum diameter	2.5 (1.3–3.8) mm
PDA length	7.1 (2.6–12.4) mm
Device	
AVP II	32 (73%)
ADO II-AS (Piccolo)	12 (27%)
Device:PDA minimal diameter ratio	1.6 (1.0–2.6)
Imaging follow-up period	26.5 (1–75) months

Data expressed as median (range), or number (%)

*AVP II* Amplatzer Vascular Plug II, *ADO II* Amplatzer Ductal Occluder II, *ADO II-AS* Amplatzer Ductal Occluder II Additional Sizes (Piccolo)

No procedure-related deaths, arrhythmias, blood transfusions, bloodstream infections, or limb ischemia were observed. Notable procedural complications occurred in 3 patients: one intraprocedural device embolization (3 mm AVP II) successfully retrieved with successful placement of a second larger device (4 mm AVP II); one intraprocedural device malposition after release resulting in DAO obstruction requiring snare-assisted device repositioning (from both the arterial and venous side); and one patient who developed transient hemolysis from a small residual PDA shunt (which spontaneously resolved). No patient had residual PDA shunting at latest follow-up. Two late deaths, unrelated to the procedure, occurred 2 and 5 months after TCPC resulting from complications of extreme prematurity.

### Left Pulmonary Artery

**Fig. 1:**
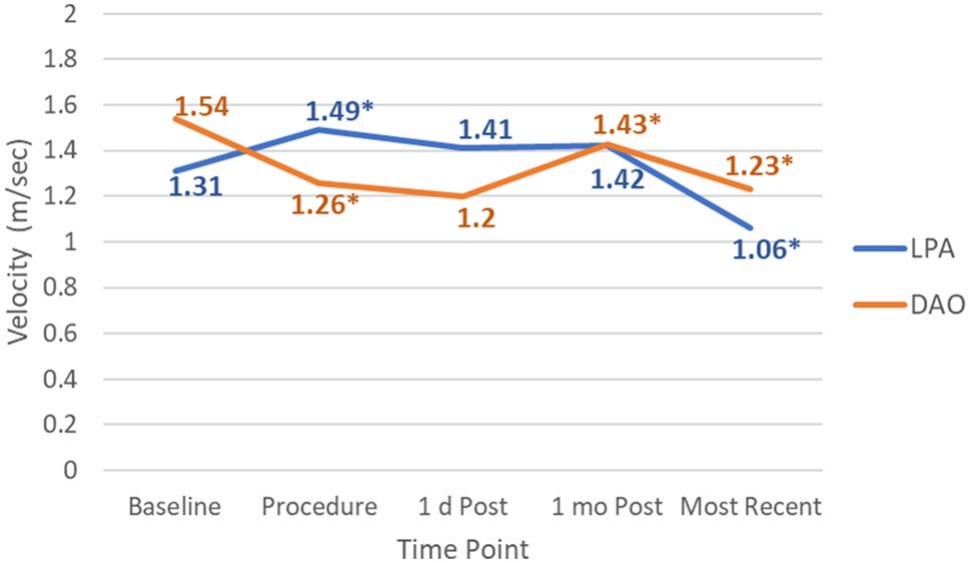
LPA and DAO velocity trends over time. Values express average LPA and DAO peak velocities for the study cohort at each imaging time point. *p < 0.01, refers to a statistically significant change in velocity as compared to the immediately preceding time point in the same vessel

Figure 1 summarizes Doppler velocity trends for the LPA and DAO over time. Peak LPA velocities generally showed an immediate increase post device placement, although the average velocity (1.49 m/s) stayed within normal limits. This remained stable during the first month post-TCPC after which velocities decreased through latest follow-up. The post device increase and remaining LPA velocity trends were congruent across both the AVP II and Piccolo devices.

**Fig. 2:**
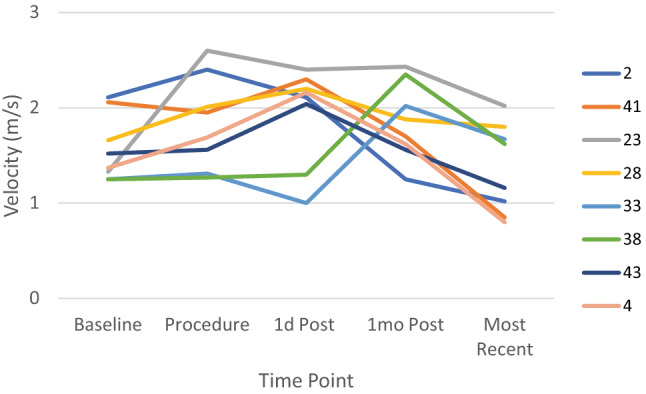
Left pulmonary artery abnormal velocities. Data represent all patients who had an abnormal peak LPA velocity (> 2 m/s) (n = 8) at any time point before or after TCPC

Two patients (Fig. 2, pt # 2 and 41) had mildly increased velocities (2.1 m/s) prior to device implantation, with no apparent LPA hypoplasia or focal stenosis. Following device implantation, 6 patients (14%) including the two mentioned above (pt # 2, 4, 23, 28, 41, 43), had elevated LPA velocities (ranging from 2 to 2.6 m/s). Among these, with the exception of pt # 23 (who had the highest peak LPA velocity (2.6 m/s) and who developed true LPA stenosis), all others had LPA velocities return to normal by 1 month. Two patients (pt # 33, 38) who had normal velocities early after TCPC developed mildly elevated LPA velocities at 1 month, although both demonstrated normal velocities and flow with no LPA stenosis at latest follow-up.

One patient in our cohort (above mentioned pt # 23, and previously reported [14]) developed device-related LPA stenosis requiring stent placement 3 months post-procedure. At the time of TCPC this infant was critically ill with a large ductus that failed multiple attempts at closure with a 4 mm AVP II. A 6 mm AVP II was implanted and a peak LPA velocity of 2.6 m/s was noted at the end of the procedure with a high suspicion for LPA stenosis; however, in light of the patient’s overall clinical status the decision was made to leave the device in place. Although the child showed significant clinical improvement following TCPC, the peak LPA velocity increased to 3.5 m/s over the ensuing 3 months and a nuclear lung perfusion scan showed 15% flow to the left lung. The patient underwent successful LPA stent placement at that time and required one subsequent stent re-dilation to accommodate somatic growth. At latest follow-up 3 years after TCPC this patient is clinically well with LPA peak velocity of 2.0 m/s.

### Descending Aorta

**Fig. 3:**
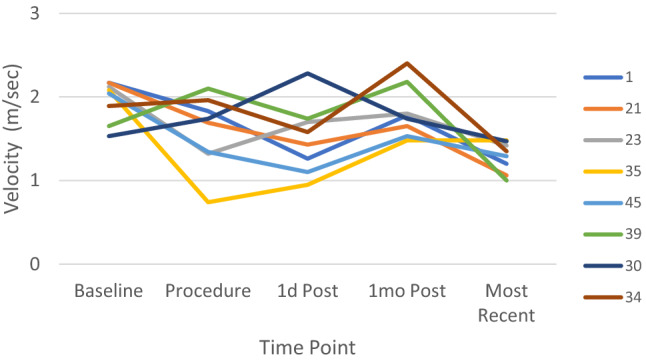
Descending aorta abnormal velocities. Data represent all patients who had an abnormal peak DAO velocity (> 2 m/s) (n = 8) at any time point before or after TCPC

DAO velocity tended to decrease immediately after device placement (Fig. 1). At 1 month follow-up, a mild increase in average velocity was observed for the group (with the mean 1.43 m/s still within normal limits) but decreased again over time through latest follow-up. No patient had a DAO velocity > 2.1 m/s at the conclusion of the procedure, including the 5 patients who had a baseline DAO velocity > 2 m/s before the procedure (Fig. 3). Mildly increased velocities (2.2–2.4 m/s) were observed in 3 patients within the first month post-TCPC (Fig. 3, pt # 30, 34, 39), but all spontaneously resolved over time. There were no cases of significant device-related CoA in our cohort.

## Discussion

This study describes our institutional experience as it relates to alterations of flow within the LPA and DAO following TCPC in EPNs. To our knowledge, this is the first study to comprehensively characterize the natural history of LPA and DAO Doppler-derived flow disturbances following TCPC in a cohort of consistently very low birth weight and premature infants at a procedural weight < 2.5 kg. As in other reported series, procedural success rate was high and the incidence of device-related LPA and DAO obstruction was low [15, 23, 26, 27].

Device-related vascular obstruction—described originally with the use of older generation devices and coils in larger children [28, 29], and more recently in younger children and infants (< 6 kg) [13, 27, 30–34]—has become a valid concern as TCPC has been extended to small premature infants. Being that this is an emerging therapy in a unique and challenging population, we believe it is important to fully examine the incidence, severity, and methods of avoiding potential complications such as device-related vascular obstruction.

### Incidence and Severity of TCPC Related Vascular Obstruction

An earlier report form our group described 24 premature infants who underwent TCPC with the AVP-II [14], with a single instance of LPA obstruction and no instance of DAO obstruction. Sathanandam et al. [26] evaluated 80 ELBW infants after TCPC with various devices and noted one case of immediate LPA stenosis after placement of a large (6 mm) AVP-II device, although the device was removed intraprocedurally with no complication. No patients in that series developed DAO obstruction. A study by Morville and Akhavi [15] using the ADO II-AS in 32 premature infants reported one case of device-related LPA stenosis 1 month post-procedure which required intervention, and no cases of DAO obstruction. A large multi-center French national registry study recently reported on successful TCPC in 101/102 infants weighing < 2.3 kg, with 3 patients developing LPA obstruction requiring treatment (2 surgery, 1 angioplasty) and 2 cases of DAO obstruction (1 requiring surgery and 1 with no evidence of clinical coarctation) [35]. Recently, Tomasulo et al. [36] reported device-related LPA Doppler flow velocity acceleration in 39% (17/44) of cases in a population weighing < 4 kg who underwent TCPC. While this relatively high incidence may be partially explained by a lower threshold for defining flow acceleration or stenosis (> 1.5 m/s), all patients with LPA acceleration in their study appeared to improve in follow-up, as did 3/4 patients with DAO flow acceleration. Finally, reporting the results of the US clinical trial of the Piccolo device, Sathanandam et al. [21] described 99/100 successful implants in premature babies < 2 kg (mean weight 1.25 kg at implant) with no instances of LPA obstruction through 6 months of follow-up and one instance of DAO obstruction.

The exception to this relatively low incidence of device-related vascular obstruction comes from a study by Chien et al. [37], who reported on 14 patients in Taiwan with an average procedural weight of 1335 g. None of the patients had vascular obstruction detected immediately during the procedure, but 8 (57%) developed LPA obstruction in follow-up (5 severe, 2 of which have required treatment). While serial echocardiography was performed, Doppler velocities were not reported. The authors felt that the most likely cause of LPA obstruction in their population was ongoing device deformation associated with ductal constriction following TCPC. This appears to be demonstrated by the fact that a post deployment-to-nominal device waist ratio of < 0.75 was predictive of occurrence of both mild and severe LPA obstruction during follow-up in their study [37]. Relative device over-sizing and a tendency for device placement favoring the MPA (potentially not fully intraductal), likely contributed to this high rate of device deformation resulting in lengthening and thus LPA stenosis. In contrast, the current study adds support to a growing body of literature, suggesting TCPC can be performed in premature neonates with a low risk of significant LPA or DAO obstruction.

### Left Pulmonary Artery Flow Patterns

In the present study, immediately following device deployment LPA velocity tended to increase relative to baseline, although on average staying within the normal range. Even in those few patients where LPA velocity increased to values above normal (2.0–2.4 m/s) during implant and/or the first 24 h post-procedure, true LPA stenosis did not develop in follow-up, and in fact velocities returned to normal pre-procedural levels over time. This is similar to other published reports, which suggest that mild intraprocedural increases in LPA velocity can be a common finding (20–39% reported in the literature) [23, 36] but largely transient, with the development of true LPA stenosis in follow-up occurring in only 0–3% of patients and even fewer requiring any intervention [13–15, 23, 26, 32].

The single patient in the current study who developed significant device-related LPA stenosis had an immediate post-implant LPA Doppler velocity > 2.5 m/s, suggesting that while post-implant LPA velocity of 2.0 m/s or slightly higher is typically benign, there may be a Doppler velocity cutoff value where stenosis is more likely to develop. The current study was not powered to definitively provide a strict periprocedural cutoff LPA velocity which is predictive of the development of clinically significant LPA stenosis, although this is an area which certainly requires further in-depth study. It would appear prudent to suggest nevertheless based upon our current level of knowledge, that an increase in LPA Doppler velocity > 2.5 m/s during TCPC should alert the operator of the possibility for the development of future LPA stenosis. This should prompt further evaluation (e.g., pulmonary artery angiography via the delivery catheter) and/or device repositioning or removal. It also should be noted that although there were no cases in the current series, there are rare reports of late LPA stenosis developing weeks after TCPC in EPNs who had reportedly normal intraprocedural Doppler profiles [15]. Based upon these reports and lack of definitive data at this time, we would advocate that every premature infant undergoing TCPC should undergo repeat examination with Doppler interrogation within 24 h, and at 1 and 3 months after the procedure, with further continued observation if Doppler velocity in follow-up is > 2 m/s or other concerns are present.

### Descending Aorta Flow Patterns

The Doppler flow velocity in the DAO typically decreased in this study following device placement. This is likely secondary to a reduction in blood volume traversing the aortic isthmus following elimination of the left-to-right ductal shunt with PDA closure. Only 2 patients experienced a rise in DAO velocity during device implant, both of whose DAO velocities returned to baseline by the next day and remained normal in long-term follow-up. While a slight increase in overall DAO velocity for the group was noted at 1 month, these values remained similar to or lower than pre-procedural baseline values, and within the normal range on average. All patients, including those who had initial pre-procedural DAO velocities > 2 m/s and those who had transient elevations of DAO velocity (as high as 2.4 m/s) during early follow-up, had normal DAO velocity at latest follow-up with no evidence for device-related coarctation. These findings support that the expectation for operators performing TCPC should be that DAO velocity will decrease during TCPC, and increases in DAO velocity should raise concern for the possible development of future CoA, prompting thoughtful consideration for device repositioning or removal.

The echocardiographic assessment of the DAO is particularly crucial during TCPC in EPNs, as arterial access has been shown to be a risk factor for arterial complications and should generally be avoided [18]. This takes pediatric interventional cardiologists out of their typical “comfort zone” for TCPC where reliance on aortic angiography and pressure measurements have long been the standard for assessing the intraprocedural risk of device-related DAO obstruction. We therefore strongly believe that a critical part of a successful TCPC program in EPNs must involve the development of seamless coordination and communication between the non-invasive imaging team and the interventional cardiologist performing the procedure.

### Potential Mechanisms of Device-Related LPA and DAO Obstruction During TCPC

**Fig. 4:**
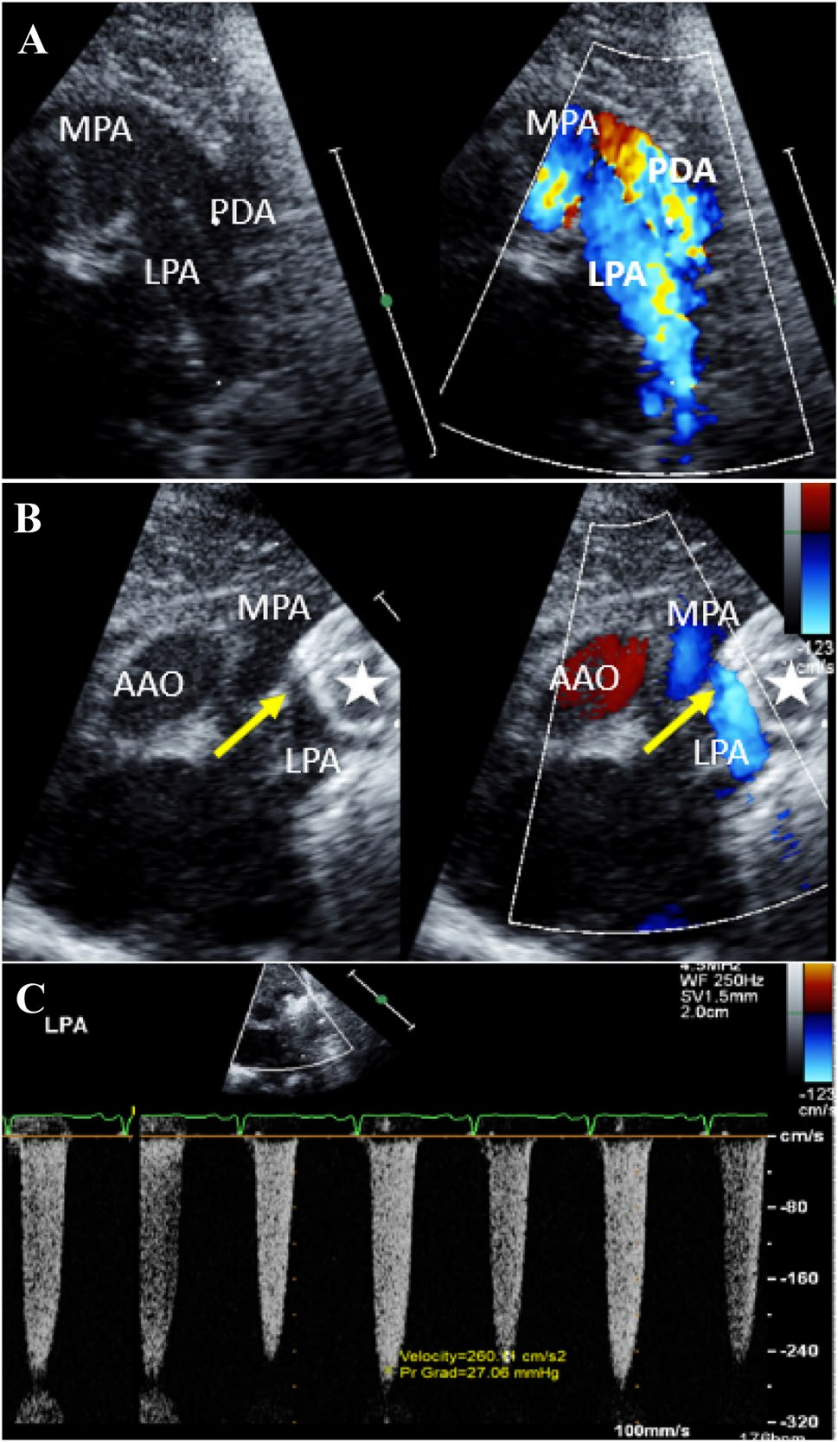
Patient who developed LPA obstruction. Echocardiographic images of the patient who developed device-related LPA stenosis. **a** 2D and color Doppler images side-by-side at baseline, before device PDA closure. A large tubular PDA is visualized with a parallel orientation to the LPA, and shunting seen across the PDA from the DAO into the distal main pulmonary artery. **b**, **c** Intraprocedural images immediately post device closure showing the proximal portion of the AVP II device (white star) compressing the proximal LPA (yellow arrow), resulting in an elevated peak LPA velocity of 2.6 m/s, despite not demonstrating a classic obstructive flow pattern of continuous diastolic flow

Increased risk for LPA or DAO obstruction has been linked to smaller patient size, larger PDA diameter and shorter ductal length (high ductal diameter:length ratio), type of ductus (e.g., window-like), and larger device [30, 36–39]. All of these factors may contribute to LPA flow disturbances and obstruction resulting from two potential mechanisms: proximal disk protrusion from the ductus directly obstructing the LPA orifice, and/or external compression of the LPA from an oversized device in the ductus exerting radial force upon it after implantation. Mild obstruction, manifested as an increased Doppler flow velocity form baseline related to either mechanism or the close confines of the neonatal pulmonary bifurcation, appears to be common. However, this generally improves over time, presumably as a function of increasing effective LPA luminal diameter with patient somatic growth. Thus, a mild intraprocedural increase in Doppler flow velocity (< 2.0–2.5 m/s) across the LPA, not associated with other evidence of LPA obstruction such as angiographic or echocardiographic 2D imaging of LPA occlusion or a diastolic runoff pattern, is likely benign in the majority of cases and may be expected to spontaneously improve. More significant intraprocedural LPA obstruction, as seen with the single patient in this series who required treatment, is typified by Doppler velocity profiles > 2.5 m/s and 2D echocardiographic evidence of LPA compromise (Fig. 4). Of note, while we have found 2.5 m/s to be a useful marker for identifying and predicting significant vascular obstruction, because there are variations in intraprocedural hemodynamics among very small and premature neonates, a nonreassuring device or LPA appearance by 2D imaging even in the absence of meeting this velocity threshold should be evaluated and addressed as necessary.

A smaller but significant number of cases of post-procedural DAO obstruction have been reported following TCPC in EPNs [23, 35, 36]. When DAO obstruction is mild and associated with only mild increases in Doppler velocity (< 2.0 m/s) it appears to be most often related to the superior aspect of the aortic disk of either the AVP II or Piccolo device extending slightly out of the aortic ductal ampulla and into the aortic lumen, despite proper device positioning and sizing. Typically this is a benign finding which self-resolves with somatic growth [36], however, there appear to be rare exceptions where cases with reportedly normal DAO flow velocities develop important DAO obstruction in the first months of follow-up [24]. Possible mechanisms include improper device positioning (too posterior), inaccurate intraprocedural echocardiographic assessment, device over-sizing, and device posterior migration as the ductus constricts from the pulmonary artery toward the DAO after closure. Further studies will be needed to gain a better understanding of this potentially dangerous complication, but given the current knowledge we feel it is prudent to recommend avoidance of device over-sizing, maintaining the aortic disk position as anteriorly within the PDA as space will allow (balancing the risk of LPA obstruction), and having a high level of suspicion for DAO obstruction when Doppler velocity increases rather than decreasing after device placement. Future efforts should continue to focus on device design and delivery improvements specifically tailored for EPNs, in attempts to further minimize complication risk and optimize outcomes after TCPC in this challenging but important patient population.

### Limitations

This was a retrospective study and therefore the limitations of such analyses apply. While we noted improvement of LPA and DAO velocities over time, and even normalization of mild flow disturbances, the sample size of patients who demonstrated abnormal velocities was relatively small. Additionally, we could not determine the precise time point at which normalization occurred as we did not analyze echocardiographic data between 1 month and latest follow up. Because our procedural echocardiographic data were taken from the conclusion of the case, it may not capture the importance of device repositioning before final release which can sometimes be utilized. Furthermore, due to a low incidence of LPA obstruction and no cases of CoA in our cohort, we were unable to conduct subgroup analysis to sufficiently evaluate other echocardiographic or angiographic parameters (e.g., PDA characteristics, device deformation, device type and size, device:ductal size ratios, etc.), which may be important to understanding the risk for development of vascular stenosis post-TCPC.

## Conclusions

TCPC can be performed successfully in extremely premature low birth weight infants with a low incidence of post-procedural LPA and DAO obstruction. In our experience, patients who develop progressive LPA obstruction typically present early and may be successfully treated with stent therapy. In this series of EPNs, late onset stenosis post-TCPC was atypical and the data suggest that in the absence of significant vascular obstruction in the early post-procedure period LPA and DAO flow disturbances are commonly mild and generally improve over time.
